# Efficacy and safety of advanced hybrid closed loop systems in children with type 1 diabetes younger than 6 years

**DOI:** 10.3389/fendo.2024.1382920

**Published:** 2024-05-21

**Authors:** Novella Rapini, Mariangela Martino, Claudia Arnaldi, Annalisa Deodati, Lilian Anagnostopoulou, Maria Elisa Amodeo, Paolo Ciampalini, Valentina Pampanini, Antonella Lorubbio, Davide Tosini, Stefano Cianfarani, Riccardo Schiaffini

**Affiliations:** ^1^ Endocrinology and Diabetes Unit, Bambino Gesù Children’s Hospital, IRCCS, Scientific Institute for Research, Hospitalization and Health Care, Rome, Italy; ^2^ PhD Program in Immunology, Molecular Medicine and Applied Biotechnologies, University of Rome ‘Tor Vergata’, Rome, Italy; ^3^ Pediatric Diabetes Unit, ASL Viterbo, Viterbo, Italy; ^4^ Department of Systems Medicine, University of Rome ‘Tor Vergata’, Rome, Italy; ^5^ Department of Women’s and Children’s Health, Karolinska Institutet, Stockholm, Sweden

**Keywords:** T1D (type 1 diabetes), CSII (continuous subcutaneous insulin infusion), children, insulin, AHCL

## Abstract

**Background:**

Tight glycemic control is essential for the normal growth and development of preschool children. The aim of our study was to evaluate the impact of advanced hybrid closed loop (AHCL) systems in a real-life setting in children younger than 6 years.

**Methods:**

We conducted a two-center prospective study. We enrolled 19 patients with a median age at disease onset of 2.6 years [interquartile range (IQR) 1.6; 4.4] and a median disease duration of 1.4 years (IQR 0.9; 2.8) who were switched to AHCL from multiple daily injections or open-loop insulin therapy and with a 6-month follow-up. Clinical data, sensor glycemic metrics, and pump settings were collected and analyzed.

**Results:**

After 6 months of follow-up, there was a significant reduction in median HbA1c (*p* = 0.0007) and glucose management indicator (*p* = 0.03). A reduction in both mild (>180 mg/dL) (*p* = 0.04) and severe (>250 mg/dL) (*p* = 0.01) hyperglycemia was observed after 1 month of auto mode, and in mild hyperglycemia, it persisted up to 6 months (*p* = 0.02). A small increase in time below range (<70 mg/dL) was observed (*p* = 0.04) without a significant difference in time <54 mg/dL (*p* = 0.73). Time in range increased significantly, reaching a 10% increment (*p* = 0.03) compared with baseline. A significant reduction in the average sensor glucose was observed (*p* = 0.01) while coefficient of glucose variability (CV%) remained stable (*p* = 0.12). No episodes of ketoacidosis or severe hypoglycemia have been recorded.

**Conclusion:**

AHCL systems are effective and safe for children younger than 6 years and should be considered as a valid therapeutic option from diabetes onset.

## Introduction

Glycemic control in preschool children is challenging and glucose management is burdened by high glycemic variability (1) due to the reduced predictability of daily activities and meals. Tight glycemic control is mandatory, as toddlers diagnosed with type 1 diabetes (T1D) are expected to be exposed to long diabetes duration to reduce complications (2, 3), minimizing at the same time the hypoglycemic risk (4). Furthermore, neuroimaging studies identified alterations particularly affecting white matter, suggesting that during toddler and preschool years, the brain is highly sensitive to metabolic disturbances (both hypo- and hyperglycemia) (5) with implications for cognitive and executive functions, intelligence quotient, delayed memory, and processing speed (6). Advanced hybrid closed loop (AHCL) devices have proven useful in improving disease management and time in range (TIR) (7), but they have been currently approved above 6 or 7 years of age with a minimum total daily dose (TDD). The aim of our study was to evaluate the impact of AHCL on glycemic control over time in children younger than 6 years in a real-life setting, compared to the previous conventional multiple daily injection (MDI) insulin therapy and open-loop continuous subcutaneous insulin infusion (CSII) treatment [with or without a sensor augmented pump (SAP)].

## Methods

We enrolled 19 pediatric patients (11 boys; 8 girls) with T1D {with a median age at disease onset of 2.6 [interquartile range (IQR) 1.6; 4.4] years and a median disease duration of 1.4 (IQR 0.9; 2.8) years} from the Bambino Gesù Children’s Hospital Diabetes Unit in Rome and at the Viterbo Pediatric Diabetes Unit, Italy, between January 2021 and June 2023. All involved families gave their consent for the use of algorithm-driven automated insulin delivery, although this therapeutic approach is currently approved in children at the age of 6 years or above with a minimum TDD of 10 units or >25 kg of weight (Tandem t:slim X2 Control-IQ) and above the age of 7 years with a minimum TDD of 8 units (Medtronic MiniMed™ 780G).

We considered the following inclusion criteria:

- age < 6 years
- diagnosis of T1D according to ISPAD guidelines (8)
- being monitored by isCGM (intermittent scanning continuous glucose monitoring) or rtCGM (real time continuous glucose monitoring) at baseline
- being on MDI insulin therapy or open-loop CSII treatment

Exclusion criteria were as follows:

- conditions or use of medications known to affect glycemic levels
- being already on the AHCL system

The study was approved by the Institutional Ethics Committees of participating centers, and all participants’ parents provided informed consent.

Weight (kg), height (cm), body mass index (BMI), glycated hemoglobin (HbA1c), and TDD in international units per body weight (IU/kg) have been evaluated. Participants’ demographic and anthropometric characteristics are shown in **Table 1**.

**Table 1:** Participants’ demographic and anthropometric characteristics.

	Median (IQR)
Number of patients	19
M/F	11/8
Duration of the disease (years)	1.4 (0.9, 2.8)
Age at disease onset (years)	2.6 (1.6, 4.4)
Age at start of AHCL (years)	4.8 (4.4, 5.5)
Weight (kg)	19 (1.2, 19.9)
Height (cm)	109 (102, 111.3)
BMI (kg/m2)	16.2 (15.6, 17.5)

Sensor glucose reading data for 30 days were collected from rtCGM, and glycemic control was evaluated, analyzing data obtained from Carelink and Glooko platforms, considering percentage of TIR (TIR%, between 70 and 180 mg/dL), time above range in mild (TAR% >180 mg/dL) and severe (TAR% >250 mg/dL) hyperglycemia, and time below range in mild (TBR% <70 mg/dL) and very low (TBR% <54 mg/dL) hypoglycemia (9). Glucose management indicator (GMI), glucose average (mg/dL), coefficient of variability (CV%), and recorded dietary carbohydrate (9) were also evaluated.

All participants reported >90% of time with sensor in use.

The last available 30 days of sensor glucose data on MDI treatment or the open-loop system, before switching to AHCL, were considered as baseline, and 30 days of sensor glucose data were considered for each of the other time points (1, 3, and 6 months) during the follow-up period.

During follow-up, nine patients used Tandem t:slim X2 in Control-IQ mode, nine used MiniMed 780G with SmartGuard mode, and one shifted from MiniMed 640G to MiniMed 670G.

The MiniMed 780G pump was initially used in manual mode for 2 weeks before switching to auto mode with a 120 mg/dL glycemic target and a 100 mg/dL glycemic target after 3 months with a mean active insulin time of 2 h.

After switching to AHCL systems, subjects were followed up for 6 months and glycemic metrics were recorded after 1, 3, and 6 months. At each visit, the family was asked whether ketoacidosis or severe hypoglycemia occurred during the reported period. Furthermore, differences in anthropometric parameters were analyzed (weight, height, and BMI) as well as the HbA1c level at the beginning and at the end of the follow-up (**Table 2**).

**Table 2:** Participants’ glycemic metrics at baseline and during follow-up.

	Baseline, median (IQR)	1 month follow-up, median (IQR)	*p*-value	3 months follow-up, median (IQR)	*p*-value	6 months follow-up, median (IQR)	*p*-value
HbA1c (mmol/mol)	56.3 (52, 62.5)					55 (44.8, 58.7)	**0.0007**
TDI (units/kg/day)	0.6 (0.5, 0.8)	0.7 (0.6, 0.8)	**0.02**	0.8 (0.7, 0.8)	**0.004**	0.6 (0.5, 0.7)	0.4
Bolus/TDI (%)	47.1 (45, 55.3)	56.6 (52, 60.2)	**0.009**	54.4 (52.2, 62.4)	0.16	54.7 (50.4, 58.8)	0.22
CHO (g)/day Recorded dietary carbohydrate	140 (112, 170)	152 (138.9, 206.6)	0.12	153 (122, 192.5)	0.43	142 (93, 167)	0.62
TBR (<54 mg/dL) %	0 (0, 1)	0 (0, 1)	0.36	0 (0, 1)	0.99	0 (0, 1)	0.73
TBR (54–70 mg/dL) %	2 (1, 3)	3 (2, 3)	0.69	2 (2, 4)	0.34	2.5 (2, 4)	**0.04**
TIR (70–180 mg/dL) %	60 (50, 59.5)	66 (72, 70.5)	**0.007**	66 (61, 70.5)	**0.03**	70 (61.3, 72.8)	**0.03**
TAR (180–250 mg/dL) %	25 (21, 27.5)	22 (19, 26)	**0.04**	22 (20.5, 24.5)	**0.01**	21 (18.3, 23.8)	**0.02**
TAR (>250 mg/dL) %	11 (6, 19.5)	9 (6, 11.5)	**0.01**	10 (6, 12)	0.09	7 (5, 9.8)	0.06
Mean glucose (mg/dL)	161.5 (150.5, 187.8)	158 (146, 165)	**0.006**	157 (150, 167.5)	**0.04**	153.5 (146, 163.8)	**0.01**
CV%	37.6 (35.8, 40)	38.1 (36.6, 40.1)	0.41	39 (35.6, 41.4)	0.29	38.3 (34.9, 41.9)	0.12
GMI%	7.2 (6.9, 7.8)	7.1 (6.8, 7.3)	**0.01**	7.1 (6.9, 7.3)	**0.03**	7 (6.8, 7.2)	**0.03**

HbA1c, glycated hemoglobin; TDI, total daily insulin; CHO, carbohydrate; TBR, time below range; TIR, time in range; TAB, time above range; CV, coefficient of variation; GMI, glucose management indicator.

All p-values are compared to baseline.

In bold the values statistically significant.

### Statistical analysis

Participants’ characteristics are reported as median and IQR for continuous variables and as absolute frequency and percentage for categorical variables.

We performed Wilcoxon signed-rank test to check whether the differences between paired data were statistically significant. We considered *p*-value below 0.05 as statistically significant.

Analyses were performed using GraphPad Prism ver. 9.00.

## Results

**Figure 1:**
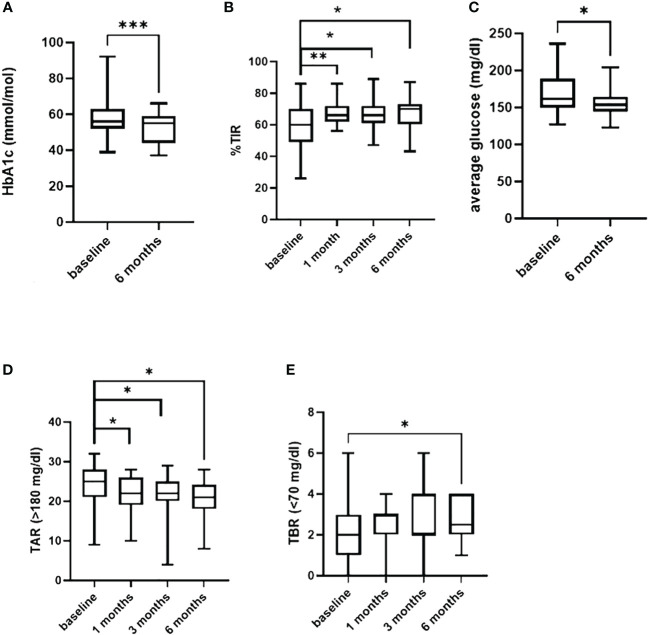
In **(A)** difference in HbA1c from baseline and 6 months of follow-up. Difference in TIR, in TAR (>180 mg/dl) and in TBR (<70 mg/dl) from baseline and 1-3-6 months follow-up are shown in **(B, D, E)**. In **(C)** difference in the average of sensor glucose. *=p<0.05; **=p<0.001; ***=p<0.0001.

After 6 months of follow-up with the AHCL systems, there was a significant reduction in both median HbA1c from 56.3 (52, 62.5) to 55 (44.8, 58.7) mmol/mol (**Figure 1A**) (*p* = 0.0007) and median GMI from 7.2 (6.9, 7.8) to 7 (6.8, 7.2) % (*p* = 0.03) without changes in BMI (*p* = 0.27, not shown).

Time in range (TIR%) increased significantly during the 6 months of follow-up, reaching a difference from baseline of +10% (*p* = 0.03) and +6% after the first (*p* = 0.007) and after the third (*p* = 0.03) month of use (**Figure 1B**). Insulin requirement presented a slight increment at 1 and 3 months (*p* = 0.02 and *p* = 0.004), without a significant change at the end of follow-up period compared to baseline (0.6 IU/kg/day, *p* = 0.4).

A significant reduction in the glucose average was observed, during the entire 6 months (*p* = 0.01) (**Figure 1C**).

A reduction in both mild (>180 mg/dL) (*p* = 0.04) and severe (>250 mg/dL) hyperglycemia (*p* = 0.01) was observed 1 month after AHCL systems, which persisted up to 6 months for mild hyperglycemia (*p* = 0.02) (**Figure 1D**).

Furthermore, time below range <70 mg/dL presented a small increment (*p* = 0.04) (**Figure 1E**) from baseline to 6 months without a significant difference in time <54 mg/dL (*p* = 0.73).

No significant differences were found in CV during follow-up (*p* = 0.12).

Recorded carbohydrates per day remained stable during the study period (*p* = 0.12, 0.43, and 0.62, respectively) with a significant reduction between 3 and 6 months (*p* = 0.02).

Both AHCL systems have always worked in auto mode during the observational period despite the low insulin demand (the minimum reported daily dose was 6 IU).

No episodes of ketoacidosis or severe hypoglycemia were reported during follow-up.

## Discussion

Our study, involving preschool children with T1D treated with AHCL systems for a follow-up period of 6 months, showed a +10% increment of TIR from baseline, reflecting a reduction of 2.5 h per day spent in a hyperglycemic state. Interestingly, improvement in TIR was already evident after 1 month and was sustained during the 6-month period, suggesting a precocious and stable effect of these systems on glycemic control. Furthermore, they led to a reduction in HbA1c, which was more evident for individuals with worse baseline levels.

We also observed an important reduction of time spent in hyperglycemia using AHCL systems, compared to baseline MDI or SAP therapy.

These results are relevant whereas detectable changes in brain volumes and cognitive scores in children with T1D are associated with hyperglycemic metrics (10).

Literature confirms these results. Recently, a 12-week open-label prospective study with MiniMed 780G™ in children 2–6 years old (TDD ≥8 IU/day) demonstrated that this device is also safe in this age group, improving glycemic control and reducing parental distress (11).

Similar lines of evidence were gathered from a retrospective analysis conducted by Tornese et al. on 12 children <7 years (minimum TDD 4 IU/day) with MiniMed 780G™ with SmartGuard who have been followed up for 12 months (12).

In another multicenter 13-week trial with Tandem t:slim X2 insulin pump with Control-IQ Technology, 102 children with a mean age of 4 years (TDD ≥5 IU/day) were assigned to receive an advanced hybrid closed-loop system of insulin delivery or standard care. TIR increased from 56.7% ± 18.0% to 69.3% ± 11.1% during follow-up in the closed-loop group. There was also a significant reduction of TAR and HbA1c, while TBR remained stable (13).

Consistent with these data, we did not observe a reduction of TBR. This finding could be explained by the fear of hypoglycemia in very young children, which led the caregivers to try to maintain a strict control on low glucose values. These systems, however, allow parents to become more confident, thanks to the automatic insulin delivery suspension, leading to a reduction of excessive sugar correction and, consequently, a reduction in time spent in hyperglycemia as well.

CV, above the threshold already at baseline, showed a slight increasing trend, similar to the results of Tornese et al. (12) at the end of the follow-up. The median values above the threshold could be attributed to the unpredictability of meals and activity in this age group while the increasing trend could be due to more frequent bolus performed by the pump.

Because of this variability, even if 48 h is sufficient, we decided to prolong the manual period before transitioning to auto mode with MiniMed 780G until 14 days to allow the algorithm to calculate a more precise basal rate.

The strengths of our study include the real-world setting and the evaluation of two different AHCL systems. A limitation of this 6-month study was its relatively short duration of follow-up, although it seems clear that the benefits on glycemic control persist.

Another limit is the small sample size. Our data should be confirmed and further assessed in other studies with extended follow-up periods and more participants.

## Conclusion

Our results confirm that AHCL systems are effective in improving glycemic control in preschool children as already shown in previous studies.

Furthermore, these pumps have proven to be safe tools, improving TIR and TAR, working in auto mode also with a TDD <8 IU/day.

In conclusion, AHCL systems, such as MiniMed 780G with SmartGuard and Tandem t:slim X2 with Control-IQ technology, are an effective therapeutic option for children younger than 6 years.

## Data availability statement

The original contributions presented in the study are included in the article/supplementary material. Further inquiries can be directed to the corresponding authors.

## Ethics statement

The studies involving humans were approved by Ospedale Pediatrico Bambino Gesù, Rome. The studies were conducted in accordance with the local legislation and institutional requirements. Written informed consent for participation was not required from the participants or the participants’ legal guardians/next of kin in accordance with the national legislation and institutional requirements.

## Author contributions

NR: Conceptualization, Supervision, Writing – original draft. MM: Formal analysis, Methodology, Writing – original draft. CA: Data curation, Investigation, Writing – review & editing. AD: Writing – review & editing, Supervision. LA: Data curation, Formal analysis, Writing – original draft. MA: Conceptualization, Investigation, Writing – review & editing. PC: Supervision, Writing – review & editing. VP: Conceptualization, Validation, Writing – review & editing. AL: Data curation, Writing – original draft. DT: Data curation, Writing – original draft. SC: Resources, Supervision, Writing – review & editing. RS: Conceptualization, Data curation, Resources, Supervision, Validation, Writing – review & editing.
